# A case report of rabies in a striped hyena (*Hyaena hyaena*) in Qazvin Province of Iran

**DOI:** 10.1002/vms3.1514

**Published:** 2024-06-24

**Authors:** Mehdi Fazlalipour, Nazanin Shabansalmani, Firouzeh Farahtaj, Siamak Massoudi, Mohammad Sadegh Khosravy, Rouzbeh Bashar

**Affiliations:** ^1^ WHO Collaborating Centre for Reference and Research on Rabies Pasteur Institute of Iran Tehran Iran; ^2^ Department of Environment Wildlife Diseases Group, Wildlife Bureau Tehran Iran

**Keywords:** FAT, Iran, rabies, striped hyena

## Abstract

Rabies is a fatal and zoonotic disease that remains endemic in Iran. In this article, rabies in a striped hyena (*Hyaena hyaena*) in Qazvin Province, Iran, was found when being hunted for using the genitals for traditional thoughts of the people. The fluorescent antibody technique confirmed rabies infection in the brain sample, and vaccination was done for injured hunter.

## Graphical abstract

Rabies in a striped hyena (*Hyaena hyaena*) in Qazvin, Iran, was found when being hunted. The fluorescent antibody technique, sellers and H&E staining confirmed rabies infection in the brain sample, and vaccination was done for injured hunter. The existence of false beliefs and superstitions, which made people think of hunting and improving public awareness, can reduce the number of human rabies infection.

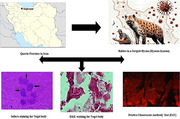

## 1 BACKGROUND

Rabies is a viral zoonosis disease caused by viruses that belong to the *Mononegavirales* order, Rhabdoviridae family and *Lyssavirus* genus. This virus can be transmitted by saliva through bites and scratches (Hooper, 2016).

Rabies is a deadly disease that can be transmitted between humans and animals. This neurotropic virus replaces and replicates in the central nervous system and then transmitted to the saliva and salivary glands. Rabies can be transmitted from domestic and/or wild animals such as dogs, cats, foxes, bats, raccoons, wolves and skunks to other animals or human, although all mammals are at risk (Dutta, 2014; Stuchin et al., 2018).

Rabies is endemic in Iran, and most of the reports about animal bites are from dogs to humans, although there have been reports about cats, foxes and rabid wolves. Hyena is a wild carnivorous animal (*Carnivora* order and Feliformia Super‐family) that generally lives in herds in the forest and feeds through hunting and scavenging. Striped hyena (*Hyaena hyaena*) is the only member of *Hyaena* genus from Hyaenidae family lives in Iran, and other species, such as spotted hyena (*Crocuta crocuta*), the brown hyena (*Parahyaena brunnea*) and the aardwolf (*Proteles cristatus*), live worldwide (Alam & Khan, 2015; Monchot & Mashkour, 2010).

The striped hyena inhabits in mountainous areas avoiding deserts, and in Iran, there is very little statistical information on the demography of these animals. Adult striped hyenas have a body length of 103–119 cm plus, and body weight ranges from 25 to 55 kg. Striped hyenas prefer very small prey such as insects, lizards, birds, rabbits and rodents for hunting (Monchot & Mashkour, 2010).

The distribution of the hyena is in most of the countries of Africa, the Indian subcontinent, throughout the Middle East and Central Asia. The distribution of this species is also widespread in Iran, and it has been reported from south to north and in most provinces of the country except West Azerbaijan, Gilan and Mazandaran (Monchot & Mashkour, 2010; Moures‐Nouri et al., 2023). Although there are limited reports of rabies in hyenas, this animal can also be considered a carrier of the disease. Hyenas are usually in the forest and not prefer to come close to humans, and for this reason, reports on the transmission of the rabies virus from hyenas to humans are limited (Boydston et al., 2003; East et al., 2001).

There is a traditional and superstitious belief that says, keeping genital organs, can capture people's hearts and increase people's affection and love. This false belief says that genital organs are unique for opening the luck (Dart, 1956; Moures‐Nouri et al., 2023).

Especially in rural areas in Iran, there is a myth that taking the female hyena's genitals attracts affection and love among individuals and can help them to get married as soon as possible. Therefore, the need for genital organs has made native people hunt hyenas.

## 2 CASE PRESENTATION

**FIGURE 1:**
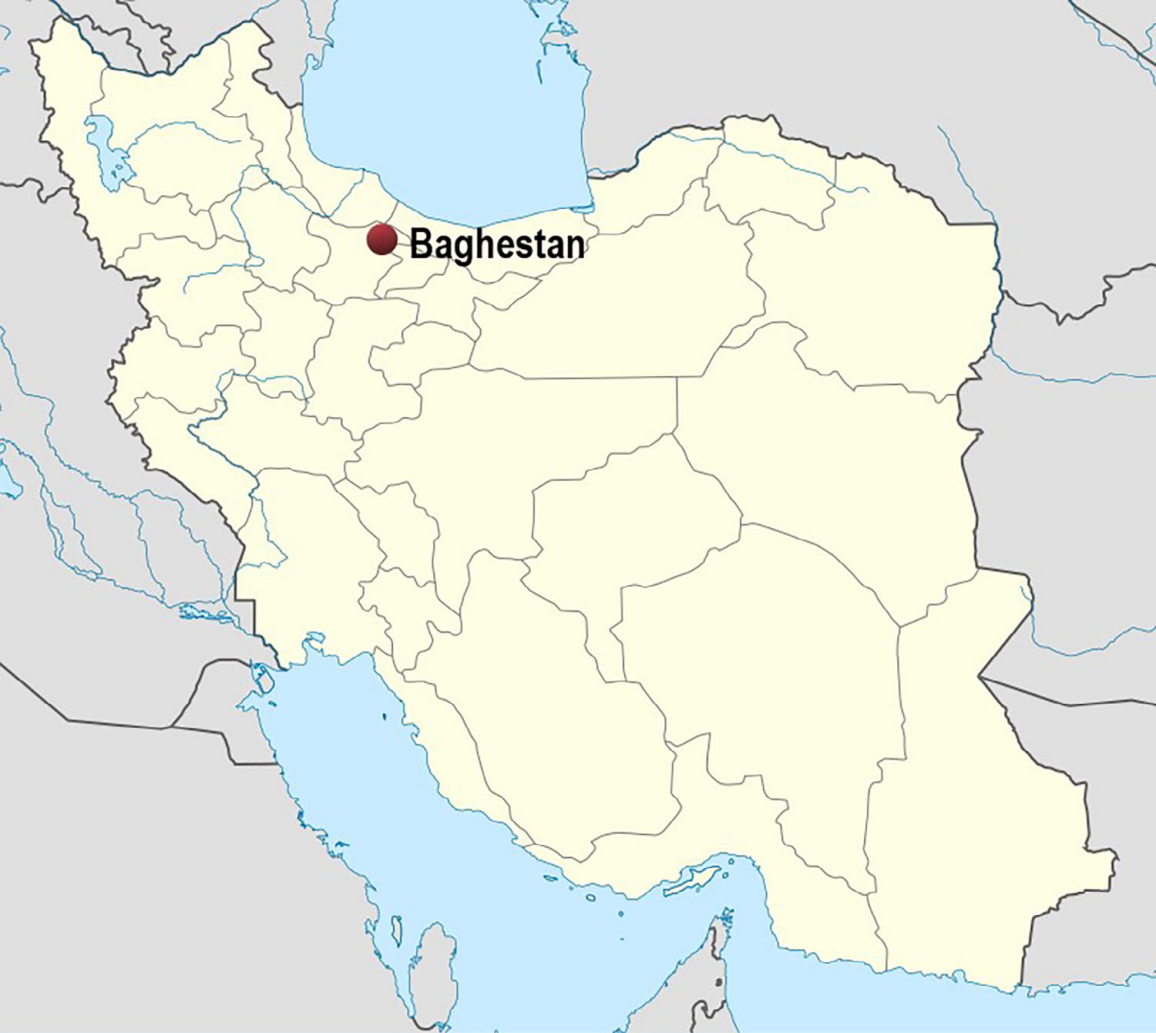
Baghdasht village in Alamut‐e Bala Rural District, Rudbar‐e Alamut District, Qazvin County, Qazvin Province, Iran.

On 7 August 2023, in Baghdasht village (Figure 1) in Alamut‐e Bala Rural District, Rudbar‐e Alamut District, Qazvin County, Qazvin Province, Iran, a hyena approached the rural residential area and the residents attacked the hyena for protection and also to remove the genitals and eventually succeeded in harming the hyena, but unfortunately when they intend to approach the hyena, the hyena bites the hand of one of the residents, which causes a serious injury to his hand. The behavioural observations such as depression and ataxia were reported.

**FIGURE 2:**
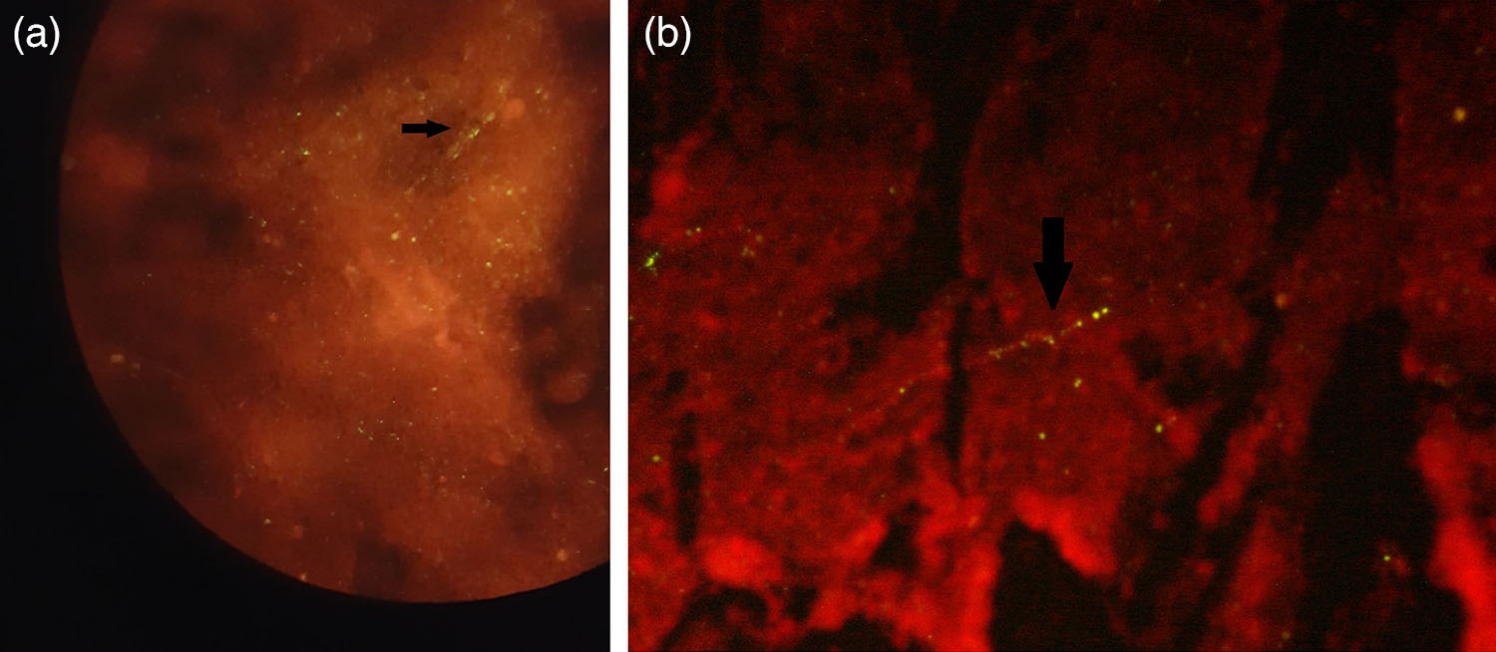
Fluorescent antibody test (FAT) positive (a and b) brain sample.

**FIGURE 3:**
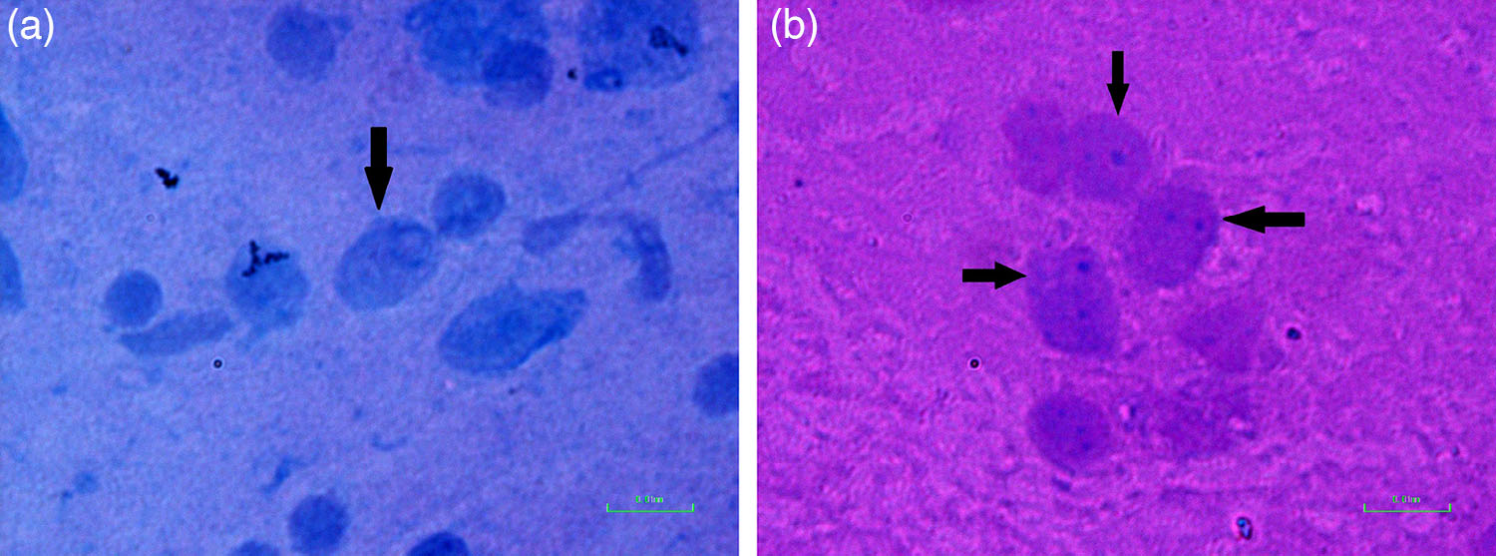
Sellers staining for Negri body detection in negative control (a) and positive hyena sample (b).

**FIGURE 4:**
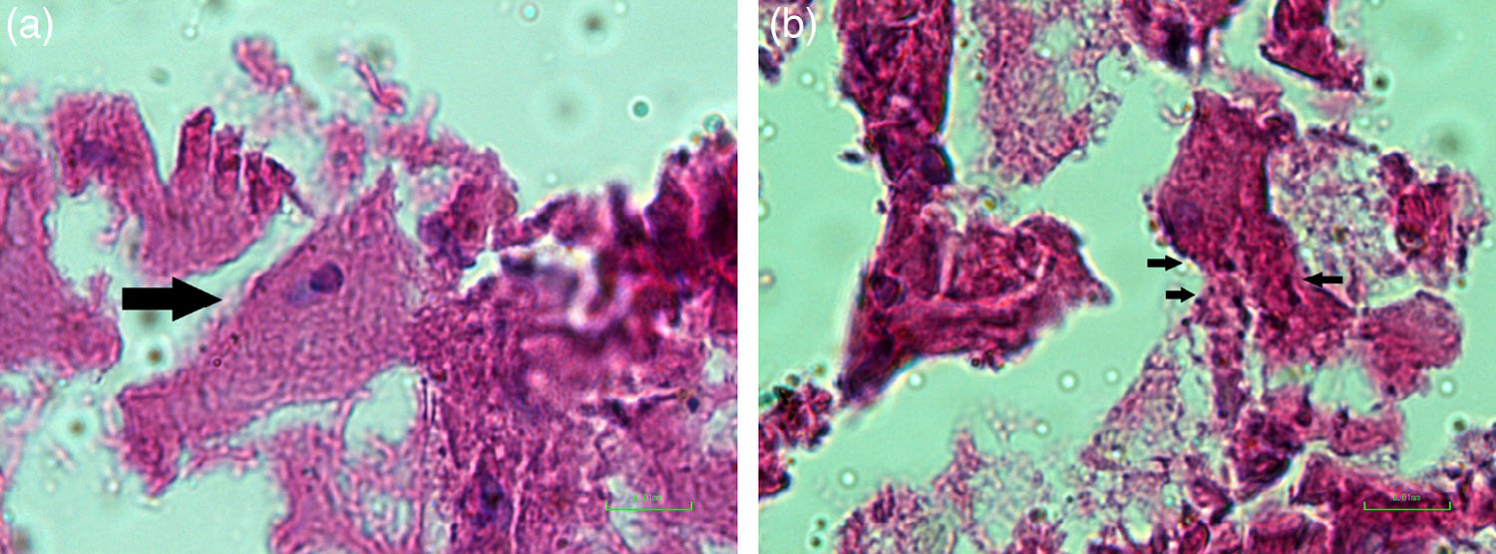
Haematoxylin and eosin (H&E) staining for Negri body detection in negative control (a) and positive hyena sample (b).

Finally, the hyena was killed, and according to the symptoms and behaviours suspected of rabies, its brain sample was refrigerated and transported (fresh on ice) to the WHO Collaborating Centre for Reference and Research on Rabies, Pasteur Institute of Iran. After performing the fluorescent antibody test (FAT) on the brain (brain stem, hypothalamus and the hippocampus), the result was reported to be positive (++ for FAT test) for the rabies virus (Figure 2). For finding Negri bodies in cytoplasm of brain cells, sellers and haematoxylin and eosin staining were performed (Figures 3 and 4) (Rupprecht et al., 2018). According to the country's protocol, the carcass of the animal is buried deep and lime is poured on the carcass, and its genitals were also taken from the residents and destroyed. Fortunately, the injured person was referred to the health care centre on time, and after washing the wound completely according to the country's protocol, a full course of rabies vaccination immediately was administrated. After informing all those who were involved in hyena hunting, they were informed and trained to carry out vaccination.

## 3 CONCLUSION

Rabies is the most important zoonotic disease which is endemic in Iran and has been reported in most provinces. Dogs, foxes and jackals as well as wolves are the most common reservoirs of the disease in Iran (Hosseini Heydarabadi et al., 2020). According to this report, it seems that the hyena was in the final phase of rabies, which caused the animal to change its behaviour and lack fear and behaviour. Aggressiveness has caused the animal to approach residential areas. Wild carnivores can be considered reservoir of rabies in the wildlife cycle and wide range of clinical symptoms can be seen.

Superstitions form a large part of the culture of human societies, have emerged over the centuries and are rooted in regional and historical conditions such as religious beliefs or the natural environment. Those who use this term imply that they have superior knowledge or evidence for their scientific, philosophical or religious beliefs. With this qualification in mind, superstitions can be roughly classified as religious, cultural and personal (Hardwick, 2023).

In this report, the existence of false beliefs and superstitions among the people made them think of hunting the animal and removing the genital organs of the hyena for love attraction and increase love affairs. It seems that increasing and/or improving public awareness, especially those who are in contact with animals or are present in forest areas through educational programmes, will reduce the number of diseases and their transmission to humans.

## AUTHOR CONTRIBUTIONS

Mehdi Fazlalipour and Nazanin Shabansalmani wrote the paper and set the data for publication and helped for FAT test. Siamak Massoudi helped to get the animal sample and collecting the local information. Firouzeh Farahtaj and Mohammad Sadegh Khosravy did the FAT test and staining of brain. Rouzbeh Bashar supervised and managed the report for publication.

## CONFLICT OF INTEREST STATEMENT

The authors declare no conflicts of interest.

## FUNDING INFORMATION

None.

## ETHICS STATEMENT

None.

## Data Availability

Data is available upon the request from corresponding author.
